# Role of MOTS-c in the regulation of bone metabolism

**DOI:** 10.3389/fphys.2023.1149120

**Published:** 2023-05-02

**Authors:** Xuejie Yi, Guangxuan Hu, Yang Yang, Jing Li, Junjie Jin, Bo Chang

**Affiliations:** ^1^ Social Science Research Center, Shenyang Sport University, Shenyang, Liaoning, China; ^2^ School of Sports Health, Shenyang Sport University, Shenyang, Liaoning, China; ^3^ School of Kinesiology, Shanghai University of Sport, Shanghai, China; ^4^ School of Physical Education, Liaoning Normal University, Dalian, Liaoning, China

**Keywords:** MOTS-c, bone, osteoblast, osteoclast, metabolism, movement presentation

## Abstract

MOTS-c, a mitochondrial-derived peptide (MDP), is an essential regulatory mediator of cell protection and energy metabolism and is involved in the development of specific diseases. Recent studies have revealed that MOTS-c promotes osteoblast proliferation, differentiation, and mineralization. Furthermore, it inhibits osteoclast production and mediates the regulation of bone metabolism and bone remodeling. Exercise effectively upregulates the expression of MOTS-c, but the specific mechanism of MOTS-c regulation in bone by exercise remains unclear. Therefore, this article reviewed the distribution and function of MOTS-c in the tissue, discussed the latest research developments in the regulation of osteoblasts and osteoclasts, and proposed potential molecular mechanisms for the effect of exercise on the regulation of bone metabolism. This review provides a theoretical reference for establishing methods to prevent and treat skeletal metabolic diseases.

## 1 Introduction

The human skeleton continuously adapts to the external environment by changing its bone microstructure and mechanical strength through bone formation and bone resorption (Lupsa and Insogna, 2015). Bone formation by osteoblasts and bone resorption by osteoclasts are two important processes that regulate bone remodeling and involve the complex coordination of multiple bone marrow cell types. The disruption in the balance between bone formation and bone resorption leads to abnormalities in bone structure and function, increased bone fragility, and increased risk of fracture (Finestone and Milgrom, 2008; Milte and Crotty, 2014; Pasco et al., 2017; Loi et al., 2016). Therefore, strategies to combat skeletal fragility are essential to maintain human bone health.

The mechanisms regulating bone remodeling are more complex and involve a greater number of factors (Robling et al., 2006). A number of studies have confirmed that mechanical loading stimulation plays a key role in bone remodeling (Alford et al., 2015; Liu et al., 2022). However, recent studies have revealed that several bioactive molecules (cytokines, proteins, peptides, metabolites, and microRNAs) are also involved in the regulation of bone metabolism and bone homeostasis, and they regulate bone remodeling by signaling between the bone and other tissues in the form of paracrine and endocrine secretions (Gonzalez-Gil and Elizondo-Montemayor, 2020; Kelch et al., 2017). In 2015, the novel mitochondrial-derived peptide MOTS-c was reported to play a role in regulating metabolic homeostasis in the body, as well as mediating skeletal muscle response to exercise. Previous studies had focused on its role in energy homeostasis, insulin resistance, and aging (Lee et al., 2015; Reynolds et al., 2021; Mohtashmi et al., 2022). Recent studies have shown that MOTS-c plays a role in regulating bone formation and resorption, and it has been hypothesized to play an important regulatory role in bone remodeling in exercise (Che et al., 2019; Weng et al., 2019). However, its mechanism of action is still being explored. Therefore, this review summarized the potential mechanisms involved in the regulation of bone metabolism by MOTS-c and further explored the possible mechanisms involved in the regulation of bone metabolism by exercise, with a view to providing a theoretical basis for subsequent studies.

## 2 Overview of tissue distribution and function of MOTS-c

Mitochondria are essential components of eukaryotic cells and are organelles for cellular energy production. 90% of the energy consumed by mammalian cells is from the mitochondria (Rolfe and Brown, 1997). It is also involved in the regulation of immune and inflammatory responses, protein homeostasis, apoptosis, stress, and adaptive responses (Lee et al., 2015). To perform these functions, mitochondria engage in complex communication links with the nuclear genome, other organelles, and, potentially, neighboring cells (Mottis et al., 2019). However, the molecular mechanisms controlling these signaling pathways are yet to be elucidated. Mitochondria, unlike other organelles in mammalian cells, have a small amount of their own DNA, called mitochondrial DNA, which is independent of the nuclear chromosomal DNA and whose short open reading frames encode small bioactive peptides, named mitochondrial-derived polypeptides (MDPs). The discovery of MDPs has opened up new areas of research, as in addition to their biological functions in cytoprotection and metabolism, each performs its own unique biological function as a possible messenger for mitochondrial function. The mitochondrial open reading frame of the 12S rRNA type-c (MOTS-c) was the second MDP discovered. It consists of 51 base pairs with a Kozak sequence (Harhay et al., 2005) and has a molecular weight of 2174.7 Da (Nashine and Kenney, 2020). MOTS-c exhibits a protein sequence of Met-Arg-Trp-Gln-Glu-Met-Gly-Tyr-Ile-Phe-Tyr-Pro-Arg-Lys-Leu-Arg, it is found mainly in muscles, white fat, and brown fat. However, it is also detected in peripheral blood.

MOTS-c is involved in metabolic regulation at the cellular and organismal levels through autocrine and paracrine secretions (Miller et al., 2020). Studies have shown that MOTS-c is expressed in the blood, brain, liver, heart, and testes (Harhay et al., 2005; Lee et al., 2015). However, it performs different biological functions in different tissues and organs. In skeletal muscle, MOTS-c increases glucose utilization and enhances insulin sensitivity, consequently regulating glucose homeostasis (Lee et al., 2015). In adipose tissue, MOTS-c increases fatty acid oxidation and white adipose lipid metabolism, activating brown adipose tissue and ultimately achieving lipid metabolic homeostasis (Lu H. et al., 2019; Li et al., 2019). Additionally, MOTS-c plays an essential role in regulating lipid accumulation in the liver by reducing hepatic fat accumulation and hepatic glucose production (Lee et al., 2015). MOTS-c acts centrally to improve cognitive memory function (Jiang et al., 2021). Furthermore, MOTS-c also improves myocardial mechanical efficiency and systolic and diastolic cardiac function (Yuan et al., 2021).

Recent studies have demonstrated a direct hormonal link between bone remodeling, food intake, and glucose metabolism, and the involvement of MOTS-c in bone metabolism (Lee et al., 2015; Ducy, 2021). These studies demonstrated that MOTS-c promotes osteoblast proliferation, differentiation, and mineralization (Hu and Chen, 2018; Che et al., 2019; Weng et al., 2019). Furthermore, it inhibits the osteoclastic differentiation involved in the regulation of bone metabolism and remodeling and possesses anti-osteoporotic effects (Ming et al., 2016; Lu Y. et al., 2019). Therefore, exploring the potential application of MOTS-c as a valuable anti-osteoporosis treatment strategy to promote bone health is promising.

## 3 Regulatory role of MOTS-c in bone metabolism and related mechanisms

### 3.1 Overview of the physiology of bone tissue

Bone tissue has several functions, including support, protection, hematopoiesis, and maintenance of the mineral balance in the body (Robling et al., 2006). The development of bone tissue is characterized by the process of bone growth and thickening. During bone development, the growth and thickening of bone constitutes the process of bone modeling, while the renewal of bone units constitutes the process of bone remodeling. Modeling is a dynamic and constructive process that ceases in adulthood, whereas remodeling is a continuous restorative process that continues throughout life (Teti, 2011). Both modeling and remodeling are essential to maintain the skeleton. Further, close crosstalk between osteoblasts and osteoclasts is required to maintain bone health and mechanical integrity (Teti, 2011; Alford et al., 2015). Osteoblasts are located on the bone surface and are rectangular cells that differentiate from bone marrow mesenchymal stem cells (BMSCs). Although osteoblasts account for only 4%–6% of total bone cells, they play an essential role in promoting bone formation (Capulli et al., 2014). The osteoblasts have an abundance of rough surfaces and rough endoplasmic reticulum and golgi apparatus, as well as a variety of secretory vesicles that continuously secrete osteoid into the bone matrix (Cullinane, 2002). Osteoblasts are a key component of bone metabolism, reinforcing bone structure and enhancing mechanical properties. Osteoclasts are the only bone resorbing cells in bone metabolism and originate from the hematopoietic stem cells of the bone marrow, mainly located on the bone surface and in the Howship sockets. If osteoclasts are hyperfunctional, they can destroy bone structure and result in osteoporosis (Cappariello et al., 2014). The dynamic balance between bone formation and bone resorption is the key to healthy bones. However, the mechanisms are complex and previous studies have focused on mechanical forces and bioactive factors secreted by the bone tissue cells themselves (Wawrzyniak and Balawender, 2022). In recent years, it has been found that mitochondria are a key component of the bone tissue and that mitochondria-derived peptides play a role in bone metabolism, in addition to their role in regulating metabolic homeostasis (Guo et al., 2003; Lee et al., 2015; Cobb et al., 2016). The role of MOTS-c in bone remodeling and its potential mechanisms are reviewed and summarized below.

### 3.2 Potential mechanisms of MOTS-c involvement in bone metabolism in osteoblasts

#### 3.2.1 Role of MOTS-c in TGF-β-mediated osteoblasts

MOTS-c has been shown to promote osteoblast differentiation. Hu and Chen (2018) showed that MOTS-c increased the expression of alkaline phosphatase (ALP), a marker of early osteoblast maturation, bone gamma-carboxyglutamic-acid-containing proteins (BGLAP), and Runx2, a transcription factor important for osteoblast differentiation, using the CCK-8 assay compared to negative controls. This suggests that MOTS-c can promote osteogenic differentiation. Furthermore, in a mechanistic study in a polymer compound model, a 150-μL (1 mM) dose of MOTS-c once daily was able to significantly rescue bone loss by local injection into the area of ultra-high molecular weight polyethylene particle (UHMWPE) implantation, protecting the bone mass (Yan et al., 2019). The above experimental studies have demonstrated the role of MOTS-c in bone formation and osteoprotection. The pathway through which MOTS-c mediates the promotion of osteogenic differentiation deserves further investigation.

Among the regulators of bone metabolism, transforming growth factor TGF-β is a multifunctional protein that can play a key role in cell proliferation, differentiation, apoptosis, and related tissue development through an autocrine/paracrine/endocrine approach (Fleisch et al., 2006). Numerous experiments have shown that TGF-β promotes early differentiation and matrix production in osteogenic progenitor cells, while inhibiting late differentiation and matrix mineralization. In the skeleton, members of the TGF-β family are involved in cellular activity and metabolism during osteogenesis by stimulating BMSCs (Janssens et al., 2005). Given the importance of TGF-β signaling in bone remodeling, particularly as a coupling factor between bone resorption and formation, Hu and Chen (2018) speculated that members of the TGF-β family might be involved in the regulation of bone metabolism together with MOTS-c. Using a CCK8 assay, they found that the highest dose of MOTS-c intervention that did not affect cell proliferation was 1.0 μm. Moreover, the expression of TGF-β1 and TGF-β2 was significantly increased after 7 days of osteogenic differentiation of BMSCs after MOTS-c intervention (Hu and Chen, 2018). Notably, this study significantly reduced the protein expression of Runx2, and ALP, after silencing TGF-β1. This suggests that silencing of TGF-β1 by MOTS-c treatment significantly inhibited the osteogenic differentiation of BMSCs. Similar results were obtained when TGF-β was silenced in hFOB1.19 cells (Che et al., 2019). This suggests a close relationship between MOTS-c and TGF-β.

In addition, TGF-β has a typical transduction function in bone. TGF-β signaling is first mediated across the plasma membrane through the formation of heterodimeric complexes of specific type I and type II serine/threonine kinase receptors. The receptors are then phosphorylated upon activation of a specific type II receptor. Activated type I receptors initiate intracellular signaling via phosphorylation of the specific Smad protein R-Smads (Chen et al., 2012). Smad is a common mediator of TGF-β signaling and plays an important role in coupling bone formation and bone resorption and in maintaining normal postnatal bone homeostasis. In particular, signaling crosstalk between the TGF-β and Smad signaling pathways appears to be critical for MOTS-c to promote osteogenic differentiation and deserves more in-depth investigation.

#### 3.2.2 Role of MOTS-c in osteoblasts mediated via the TGF-β/Smad pathway

Two factors, TGF-β and Smad, act synergistically to participate in the regulation of osteoblasts, directly or indirectly promoting osteoblast differentiation, inhibiting bone resorption, and promoting bone formation. Smad, a key intracellular effector of TGF-β, is an intracellular signaling protein that has been identified in invertebrates by genetic screening in recent years. Smad mediates the osteoblast TGF-β and osteoclast bone morphogenetic protein signaling pathways and therefore plays a critical role in the regulation of bone remodeling (Iwasaki et al., 2018). Both Smad-dependent and non-dependent pathways greatly promote osteoblast differentiation and bone formation (Chen et al., 2012) and can regulate mesenchymal differentiation by mediating the Runx2 gene (Lee et al., 2002). Runx2 has an essential role in osteoblast differentiation (Chen et al., 2014). It directly regulates the expression of several osteogenic genes, including type I collagen, ALP, bone bridging protein, osteonectin and osteocalcin (Iwasaki et al., 2018). Therefore, Smad can regulate bone metabolism by regulating the expression of Runx2, a key osteogenic factor, through both dependent and non-dependent pathways to influence the differentiation and survival of osteoblasts. Hu and Chen (2018) confirmed that MOTS-c promotes bone marrow MSC differentiation toward osteogenesis via the TGF-β/SMAD pathway. In another study, the role of MOTS-c in TGF-β/Smad pathway-mediated osteogenesis was again validated by silencing TGF-β and Smad and observing changes in the expression of downstream osteogenic genes (Che et al., 2019). Studies on the pathway of MOTS-c in the direction of osteogenesis are limited. Hence, the exact mechanism of whether this pathway can affect downstream factors and promote osteogenic differentiation needs to be further evaluated.

#### 3.2.3 Role of MOTS-c in the synthesis of type I collagen by osteoblasts

In addition, MOTS-c has been shown to promote the expression of type I collagen secreted by osteoblasts. Type I collagen is the main bone matrix protein, accounting for 90% of bone matrix proteins (Szulc, 2018). Type I collagen consists of two α1 chains and one α2 chain, the gene encoding the α1 peptide chain is COL1A1 and the gene encoding the α2 peptide chain is COL1A2 (Lu Y. et al., 2019). TGF-β is thought to be the most direct cytokine affecting the synthesis and breakdown of type I collagen. Che et al. (2019) demonstrated that MOTS-c not only promoted the protein synthesis of TGF-β and Smad7 in osteoblasts, but also promoted the expression of type I collagen-related genes COL1A1 and COL1A2. This experiment was first performed by treating the osteoblast cell line hFOB1.19 with different concentrations of MOTS-c. The survival rate of the cells increased after 24 and 48 h of incubation, respectively, indicating that MOTS-c could promote the proliferation of osteoblast cell line hFOB1.19. Moreover, the expression of COL1A1 and COL1A2 mRNA in hFOB1.19 cells treated with MOTS-c increased in a dose-dependent manner. This experiment further demonstrated the upstream and downstream relationships of this pathway, again suggesting that MOTS-c can promote osteoblast type I collagen synthesis and osteogenic differentiation through the TGF-β/Smad pathway. In this experiment, hFOB1.19 cells were divided into control, MOTS-c, and MOTS-c + TGF-β inhibitor groups. After 24 h of culture, the expression of COL1A1 and COL1A2 in the MOTS-c group was significantly higher than that in the control group, and it was reduced in the TGF-β inhibitor group. Similarly, when hFOB1.19 cells were treated differently with the MOTS-c or MOTS-c + Smad7 inhibitor group, the results showed that COL1A1 and COL1A2 were significantly upregulated in the MOTS-c group compared to the control group, and the Smad7 inhibitor could partially reduce the expression of COL1A1 and COL1A2. The above results suggest that MOTS-c promotes osteoblast type I collagen synthesis through the TGF-β/Smad pathway.

### 3.3 Potential mechanisms of MOTS-c involvement in bone metabolism in osteoclasts

#### 3.3.1 Role of MOTS-c in RANKL-mediated osteoclasts

The mitochondria-derived peptide MOTS-c was also found to be important in osteoclasts, the precursor cells of bone marrow monocytes and macrophages, both of which form multinucleated osteoclasts through fusion with the bone tissue microenvironment (Scott et al., 2016). Both osteoclast formation and function are regulated by macrophage colony-stimulating factor (M-CSF), NF-κB ligand receptor activator (RANKL), and cytokines (Boyce, 2013). M-CSF is derived from osteoclast precursor mesenchymal cells and RANKL is derived from osteoblasts, osteocytes, and mesenchymal cells. M-CSF drives downstream signaling through RANKL to promote osteoclastogenesis (Edwards and Mundy, 2011). It has been shown that MOTS-c inhibits RANKL-induced osteoclast-specific gene expression and osteoclast formation (Ming et al., 2016). RANKL is a type II transmembrane protein of the tumor necrosis factor (TNF) superfamily with an N-terminal region and a C-terminal receptor binding domain (Nelson et al., 2013). A number of studies have shown that RANKL plays a central role in the regulation of mature osteoclast formation (Tanaka, 2017; Takayanagi, 2021; Bassett and Williams, 2016). RANKL knockout mice (Tnfsf11^−/−^ mice) exhibit more severe osteoporosis than control mice (Li et al., 2000; Min et al., 2000). The lack of RANKL gene expression in mouse osteoblasts causes a significant reduction in mature osteoclasts (Nakashima et al., 2011; Xiong et al., 2011). This demonstrates the important role of RANKL signaling in osteoclastogenesis (Ono and Nakashima, 2018). In an *in vivo* study, administration of MOTS-c intraperitoneally to de-ovalized osteoporotic mice once daily for 12 weeks at a dose of 5 mg/kg, lead to an increased positive trend in several bone formation parameters, such as bone mineral density, bone volume/tissue volume, bone trabecule number, and bone trabecule thickness, indicating that the MOTS-c intervention effectively reduced bone loss. Cellular experiments were also performed to detect the effect of MOTS-c on osteoclast formation by administering different concentrations of MOTS-c and counting the number of osteoclasts. There were 15 ± 5 cells per well after 50 mM MOTS-c addition, while the control group had 105 ± 5 cells per well (Ming et al., 2016). The above findings suggest that MOTS-c intervention significantly inhibited the differentiation of bone marrow stromal cells to mature TRAP-positive multinucleated osteoblasts. And it was found that MOTS-c specifically inhibited osteoclast differentiation in a dose-dependent manner. The study further examined the expression of the osteoclast molecular markers NFATc1, TRAP, histone K, and c-Fos. In response to RANKL stimulation, the expression of all these genes was upregulated and this upregulation was inhibited by MOTS-c treatment (Ming et al., 2016).

#### 3.3.2 Role of MOTS-c in OPG/RANKL-mediated osteoclasts

In a UHMWPE model-induced osteolysis mouse model, MOTS-c was found to increase the intracellular osteoprotegerin (OPG)/RANKL ratio in osteocytes, thereby inhibiting osteoclastogenesis. OPG is a soluble circulating decoy receptor for RANKL and is secreted by cells and binds to the extracellular structural domain of RANKL, preventing RANKL from osteoclast binding (Honma et al., 2013). Indeed, mutant OPG proteins expressed in co-cultured OPG-deficient osteoblasts retain their ability to bind RANKL but lack the ability to regulate RANKL transport and could not restore osteoclast formation to wild-type levels, suggesting that osteoclastogenic capacity is at least partially regulated by OPG/RANKL (Honma et al., 2013; Honma et al., 2014). Most of the factors that induce RANKL expression also induce OPG expression, albeit to a lesser extent, but increase bone resorption (Kearns et al., 2008).

The role of MOTS-c in osteoclasts is closely related to the regulation of bone metabolism by OPG/RANKL. The effect of MOTS-c on osteoclast secretion and osteoclastogenesis was examined according to the changes in the OPG/RANKL ratio *in vivo*. The study collected osteoclast supernatants after 12 h of MOTS-c treatment, in which TRAP-positive multinucleated osteoclasts were found to be significantly inhibited. The results of the study showed that OPG expression was significantly increased but RANKL expression was decreased in the MOTS-c intervention group compared to the control group. Considering that OPG and RANKL are mainly secreted by osteoblasts to regulate bone metabolism, the inhibition of MOTS-c-induced osteoclastogenesis may be caused by an increase in the OPG/RANKL ratio. The effect of MOTS-c on OPG and RANKL expression was also associated with AMP-activated protein kinase (AMPK) activation, as the inhibition of AMPK phosphorylation downregulated OPG levels and upregulated RANKL levels. Thus, MOTS-c may regulate osteoclast secretion through the activation of AMPK (Yan et al., 2019). A previous study has shown that MOTS-c intervention significantly ameliorates bone loss in 8-week-old C57BL/6 female ovariectomized mice by inhibiting osteoclast formation in an AMPK-dependent manner to prevent ovariectomy-induced bone loss (Ming et al., 2016). As an indispensable element in cell signaling, reactive oxygen species (ROS) act as a second messenger during RANKL-induced osteoclast differentiation and activation (Kim et al., 2017). In primary bone marrow macrophages, MOTS-c may inhibit NF-κB by reducing ROS production (Chandel et al., 2000), and the expression of peroxisome proliferator-activated receptor-gamma coactivator-1 alpha (PGC-1α), a coactivator gene involved in mitochondrial biogenesis and metabolism, is significantly increased during this process. It is theorized that MOTS-c may inhibit NF-κB phosphorylation via the AMPK-PGC-1α-ROS axis, thereby affecting osteoclastogenesis (St-Pierre et al., 2006; Zhu et al., 2010; Buler et al., 2014; Yan et al., 2019). Table 1 summarizes the effects of MOTS-c on bone metabolism.

**TABLE 1:** Effect of MOTS-c on bone metabolism.

Background	Intermediate factors/pathways	Interventions	Main impacts	References
Bone formation, Bone marrow mesenchymal stem cells	TGF-β/Smad, TGF-β1, TGF-β2, and Smad7	Cellular assay, 1 μM MOTS-c induced osteogenic differentiation of bone marrow mesenchymal stem cells for 7 days and showed a significant increase in ALP, BGLAP, and Runx2 protein expression	MOTS-c promotes the differentiation of BMSCs into osteoblasts; MOTS-c significantly promotes the formation of calcified nodules of bone marrow mesenchymal stem cells	Hu and Chen (2018)
Bone formation, Bone marrow mesenchymal stem cells	FOXF1/TGF-β	In cellular assays, the relative levels of ALP, BGLAP, and Runx2 were significantly upregulated after 1 μM MOTS-c intervention and osteogenesis induction for 7 days; alkaline phosphatase activity was enhanced in BMSCs after MOTS-c treatment; and the number of mineralized nodules was greater after MOTS-c treatment	MOTS-c significantly induces osteogenic differentiation of BMSCs, thereby accelerating fracture healing	Weng et al. (2019)
Osteoblasts Type I collagen	TGF-β, SMAD7, COL1A1, COL1A2	In cellular assays, 1 μM MOTS-c treatment of hFOB1.19 cells for 1 and 3 days resulted in significant upregulation of TGF-β, Smad7, COL1A1, and COL1A2 gene expression, contributing to the synthesis of type I collagen-related genes in osteoblasts	MOTS-c promotes type I collagen synthesis by osteoblasts via the trans-TGF-β/SmAD pathway	Che et al. (2019)
osteoclasts	AMPK, RANKL, NFATc1, CTSK, c-Fos	*In vivo* study: micro-CT results following intraperitoneal injection of MOTS-c at a dose of 5 mg/kg/d showed an increase in the positive bone formation parameters BMD, BV/TV, Tb.N, and Tb.Th; *ex vivo* study: osteoclast molecular markers NFATc1, TRAP, cathepsin K, c-Fos expression increased after 10 μM MOTS-c intervention	MOTS-c has a direct inhibitory effect on osteoclasts	Ming et al., 2016
osteoclasts	OPG/RANKL	*In vivo* experiment: 150-μL/day MOTS-c *in vivo* injection; micro-CT showed an increase in the bone formation parameters BMD, BMC, BV/TV, and Tb.Th	MOTS-c inhibits osteoclast formation by regulating osteoclast OPG/RANKL secretion, and MOTS-c protects bone mass	Yan et al., 2019

## 4 Potential pathways of exercise to promote bone metabolism through MOTS-c

MOTS-c acts on multiple tissues through autocrine, paracrine, and endocrine signaling, playing a role in regulating the metabolism and steady state of the body (Lee et al., 2015). Regular exercise is a preventive and therapeutic measure for most metabolic diseases, as it increases mitochondrial activity and promotes the synthesis and secretion of endogenous MOTS-c, which in turn activates signaling pathways associated with exercise. Exogenous supplementation of MOTS-c can likewise have an exercise-like effect (Lee et al., 2015; Kim et al., 2018), thus indicating that MOTS-c may be a exercise mitochondrial factor. Exercise impacts bone metabolism, growth, and development. Regular exercise is the best way to maintain healthy bones (Warden and Fuchs, 2021; Turner and Robling, 2022). Long-term appropriate forms of exercise and appropriate exercise intensity can promote the balance between bone formation and bone resorption, increase bone trabecular alignment and density, accelerate bone mineralization, enhance bone mass, and improve bone metabolism by regulating the signaling molecules secreted by osteoblasts and osteoclasts (Fonseca et al., 2014; Mages et al., 2021; Benedetti et al., 2018). Several studies have found that exercise can affect the levels of MOTS-c and, similarly, that it regulates osteogenic differentiation and promotes bone formation (Benedetti et al., 2018; Guo et al., 2020; Chang et al., 2022; Barry and Kohrt, 2008). However, the exact signal transduction mechanism by which exercise improves bone health through MOTS-c remains unknown and needs to be further investigated.

MOTS-c acts as an “exercise mimetic,” and exogenous administration of MOTS-c can exhibit an “exercise-like” effect, therefore protecting the skeleton. In addition, MOTS-c serves as a signal for mitochondrial contact with external entities, and our group has shown that serum and skeletal muscle MOTS-c levels and mRNA levels were significantly increased in mice engaged in 8 weeks of aerobic training compared to control mice. These data strongly suggest that exercise may enhance MOTS-c activity and promote MOTS-c expression. It has been confirmed that MOTS-c could act as an effector for exercise (Guo et al., 2020). However, studies on its relationship with bone metabolism have not been reported. In one study, sedentary healthy young male volunteers exercising on bicycles had plasma samples collected before, during, and after exercise and after 4 h of rest. ELISA results revealed that the relative levels of circulating endogenous MOTS-c (i.e., individual changes based on pre-exercise values) also increased significantly during (1.6-fold) and after (1.5-fold) exercise and then returned to baseline after 4 h of rest. These results suggest that exercise induces the expression of MOTS-c (Che et al., 2019). Collectively, the studies suggest that MOTS-c may play a role in exercise-related signaling mechanisms. However, the specific mechanism of the exercise regulation of MOTS-c and bone metabolism is still unclear, and it is important to further investigate the role of MOTS-c in the regulation of bone metabolism by exercise.

Studies suggest that exercise activates AMPK (Hardie et al., 2012). AMPK is a versatile and motility-sensitive cellular energy sensor. Various upstream signals activate AMPK, notably, MOTS-c activates AMPK by increasing the intracellular levels of AICAR (an AMPK agonist) (Alis et al., 2015). Additionally, AMPK is a motility regulator (Tófolo et al., 2015; Merrill et al., 1997; Spaulding and Yan, 2022). Several studies have shown that AMPK signaling plays a role in bone metabolism (Gao et al., 2010; Shah et al., 2010; Xi et al., 2016). Therefore, AMPK is speculated to be essential in enhancing bone metabolism through exercise. However, it was found that MOTS-c activates AMPK and that MOTS-c downregulates RANKL and upregulates OPG to inhibit osteoclastogenesis (Yan et al., 2019). It has been reported that metformin and exercise can reduce osteoclastogenesis (Lee et al., 2010; Mai et al., 2011; Li et al., 2014) and studies have demonstrated that MOTS-c and metformin may share similar metabolic regulatory mechanisms (Corominas-Faja et al., 2012; Cabreiro et al., 2013). Therefore, it is hypothesized that MOTS-c and exercise have similar mechanisms and effects in regulating bone metabolism. Exercise demonstrated a dual effect, inhibiting bone resorption and stimulating bone formation. In addition, MOTS-c plays a role in enhancing locomotor activity. Young rats (CD-1) were fed a high-fat diet and treated with two doses of MOTS-c (5 and 15 mg/kg/day; intraperitoneal injection). Mice receiving a higher dose of MOTS-c demonstrated significant improvement in locomotor performance after 10 days of treatment (Che et al., 2019).

Our previous studies demonstrated that aerobic exercise of skeletal muscle activates AMPK and its downstream proliferation-activated receptor coactivator PGC-1α, thereby increasing MOTS-c expression. In addition, intraperitoneal injection of MOTS-c has the same effect as aerobic exercise, aerobic exercise and intraperitoneal MOTS-c interventions are beneficial in increasing skeletal muscle fatty acid oxidation and glucose utilization (Guo et al., 2020; Yang et al., 2021). AMPK/PGC-1α is a key component of bone metabolism. However, the exact mechanism of AMPK/PGC-1α regulation by MOTS-c in bone metabolism as a response pathway to perceived exercise stimuli is unknown. The implication of MOTS-c as evidence of feedback control of glucose and lipid metabolism by the bone to confirm the interrelationship between bone and energy metabolism, remains to be investigated. Previous studies demonstrated that exercise promotes the expression of anti-inflammatory factors and inhibits pro-inflammatory factors. Similarly, MOTS-c may inhibit NF-κB by reducing the production of ROS (Chandel et al., 2000), thereby downregulating the levels of the pro-inflammatory factors TNF-α, IL-1β, and IL-6 and upregulating the levels of the anti-inflammatory factor IL-10 (Yan et al., 2019), the effect of modulating this process by exercise is unknown.

**FIGURE 1:**
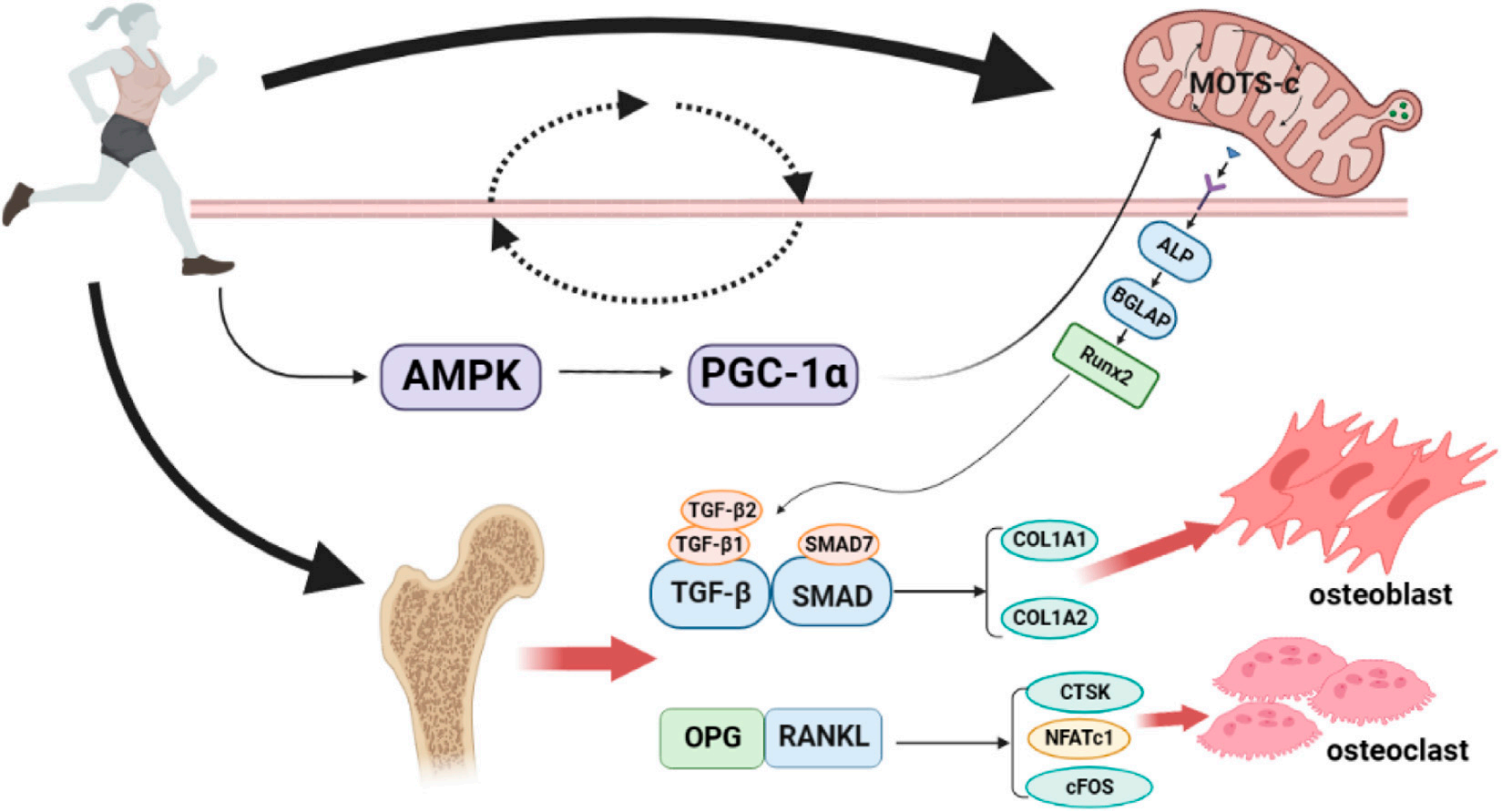
Potential pathways of exercise to promote bone metabolism through MOTS-c. Exercise can increase the expression of MOTS-c, thus activating the co-activator AMPK, resulting in an increase in PGC-1α, which in turn increases the expression of the mitochondrial-derived peptide MOTS-c, forming a loop that may regulate the expression of osteoblast/osteoclast-related genes, and ultimately, the osteoblast-osteoclast balance. AMPK, AMP-activated protein kinase; PGC-1α, Peroxisome proliferator-activated receptor-γ co-activator-1α; ALP, Alkaline phosphatase; BGLAP, bone γ-carboxyglutamate protein; Runx2, Runt-related transcription factor 2; TGF-β1, transforming growth factor-β1; TGF-β2, transforming growth factor-β2; SMAD7, Small mothers against decapentaplegic7; COL1A1, Collagen type I alpha 1; COL1A2, Collagen type I alpha 2; OPG, Osteoprotegerin; RANKL, nuclear factor-κB ligand; CTSK, cathepsin K; NFATc1, activated T Cells c1; cFOS, pleiotropic transcription factor. Produced using Biorender (biorender.com).

In summary, exercise can increase the expression of MOTS-c, which activates the coactivator AMPK, consequently increasing the PGC-1α. The cascade results in increased mitochondria-derived peptide MOTS-c expression, thus forming a loop that may regulate the expression of osteoblast or osteoclast-related genes. Furthermore, the genes expressed ultimately regulate osteoblast–osteoclast homeostasis and play an essential regulatory role in the process of exercise thereby improving bone metabolism in the body. Figure 1 provides an overview of the molecular mechanisms of potential pathways through which exercise promotes bone metabolism through MOTS-c.

## 5 Summary and outlook

An increasing number of studies have confirmed that MOTS-c plays a vital role in bone metabolism and is instrumental in stimulating osteoblast proliferation, differentiation, and mineralization and inhibiting osteoblast apoptosis. Furthermore, MOTS-c inhibits the production of osteoclasts. There are several concerns in this field: firstly, despite the association of the mitochondria-derived peptide MOTS-c with bone metabolism and current studies confirming the effects of MOTS-c intervention, the role and mechanism of endogenous skeletal MOTS-c in regulating bone metabolism have not been evaluated. Secondly, current studies on the effect of MOTS-c on bone metabolism were conducted in normal physiological states, and no relevant studies have been conducted in the pathological state. Therefore, outcomes in pathological states are unknown. Thirdly, the mechanism of action of MOTS-c on bone metabolism has been under-investigated, and most studies focus on the *ex vivo* mechanism, and fewer on the *in vivo* mechanism.

Further exploration of the direct targets or receptors of the mitochondria-derived peptide MOTS-c in regulating bone metabolism, upstream and downstream signaling, and other molecular mechanisms is required. Finally, regular exercise promotes bone growth and development and maintains bone health. However, it is unclear whether exercise affects bone metabolism by regulating the MOTS-c signaling pathway. Therefore, it is crucial to investigate the mechanisms of MOTS-c in osteogenesis, osteolysis, and bone remodeling to reveal the role of MOTS-c in bone metabolism. These investigations will provide new research directions and therapeutic targets for diagnosing, treating, and preventing skeletal metabolic diseases.

The role of MOTS-c in improving metabolism could benefit patients with metabolic syndrome. Bones and skeletal muscles are essential components of the locomotor system originating from the mesoderm, differentiated from mesenchymal stem cells, and are therefore inextricably linked. Future research could focus on: 1) the role of MOTS-c in the mechanical regulation and chemical signaling dialogue between muscle and bone; 2) the mechanism of exercise-induced MOTS-c expression in the musculoskeletal system to treat and prevent osteoporosis.
